# A Mix of Old British and Modern European Breeds: Genomic Prediction of Breed Composition of Smallholder Pigs in Uganda

**DOI:** 10.3389/fgene.2021.676047

**Published:** 2021-06-23

**Authors:** Brian Martin Babigumira, Johann Sölkner, Gábor Mészáros, Christina Pfeiffer, Craig R. G. Lewis, Emily Ouma, Maria Wurzinger, Karen Marshall

**Affiliations:** ^1^Department of Sustainable Agricultural Systems, Division of Livestock Sciences, University of Natural Resources and Life Sciences, Vienna, Austria; ^2^International Livestock Research Institute, Kampala, Uganda; ^3^PIG Austria GmbH, Steinhaus, Wels, Austria; ^4^Pig Improvement Company, Barcelona, Spain; ^5^International Livestock Research Institute, Nairobi, Kenya

**Keywords:** pig, diversity, admixture, Uganda, SNP, genomic, breed

## Abstract

Pig herds in Africa comprise genotypes ranging from local ecotypes to commercial breeds. Many animals are composites of these two types and the best levels of crossbreeding for particular production systems are largely unknown. These pigs are managed without structured breeding programs and inbreeding is potentially limiting. The objective of this study was to quantify ancestry contributions and inbreeding levels in a population of smallholder pigs in Uganda. The study was set in the districts of Hoima and Kamuli in Uganda and involved 422 pigs. Pig hair samples were taken from adult and growing pigs in the framework of a longitudinal study investigating productivity and profitability of smallholder pig production. The samples were genotyped using the porcine GeneSeek Genomic Profiler (GGP) 50K SNP Chip. The SNP data was analyzed to infer breed ancestry and autozygosity of the Uganda pigs. The results showed that exotic breeds (modern European and old British) contributed an average of 22.8% with a range of 2–50% while “local” blood contributed 69.2% (36.9–95.2%) to the ancestry of the pigs. Runs of homozygosity (ROH) greater than 2 megabase (Mb) quantified the average genomic inbreeding coefficient of the pigs as 0.043. The scarcity of long ROH indicated low recent inbreeding. We conclude that the genomic background of the pig population in the study is a mix of old British and modern pig ancestries. Best levels of admixture for smallholder pigs are yet to be determined, by linking genotypes and phenotypic records.

## Introduction

The pig (*Sus scrofa domesticus*), an even toed ungulate and a member of the genus Sus, was domesticated from its ancestor, the wild boar (*Sus scrofa scrofa*) in multiple domestication centers including the Near East, Europe, China and South-east Asia, about 9,000 years ago (Rothschild and Ruvinsky, 2011; Groenen et al., 2012). Wild boar (*Sus scrofa algira*) also inhabits North Africa (Rothschild and Ruvinsky, 2011). Since its domestication, the pig has been genetically improved into several specialized breeds through traditional and marker assisted selective breeding (Dekkers, 2004; SanCristobal et al., 2006; Mote and Rothschild, 2020). Such work is notable for European breeds such as the Pietrain that has been intensively selected for muscle development (Amaral et al., 2011). The Landrace breed originated from British foundation stock imported to Denmark and selected for leanness and fast growth. Commercial breeds such as the Large White, Berkshire, and Hampshire were developed from crossbreeding old British and Asian pigs (White, 2011; Amills et al., 2013). Iberian pigs (Toro et al., 2008) were exported during the colonization of the Americas and contributed to development of the Duroc (Jones, 1998). Its adaptive attributes and importance as a source of meat have contributed to the global distribution of the pig (Orr and Shen, 2006). Notably, sub-Saharan Africa is not within the native range of wild boar and no archeological or genetic evidence points to a domestications event there (Ramirez et al., 2009). The origin of pigs in East Africa is traced to both the pre- and colonial eras (Blench, 2000, 2008, 2010; Ramirez et al., 2009). Indian Ocean trade and eventual European settlement have also been associated with the introduction of Asian and European pig breeds to East Africa (Boivin et al., 2013).

Pig production is an important livelihood source for smallholder farms managed under low input systems in African countries, for example Nigeria, Uganda and Malawi. Uganda is an East African inland country linked to the Indian Ocean through Kenya (east) or Tanzania (south-east). Pigs in Uganda are represented by domestic pigs (*Sus scrofa domesticus*) and the wild suids including the Giant forest hog (*Hylochoerus meinertzhageni*), Warthog (*Phacochoerus aethiopicus*), and Bush pig (*Potamochoerus porcus*) (Ghiglieri et al., 1982; Reyna-Hurtado et al., 2014). In the mid-19th century, Britain colonized Kenya and Uganda while Germany colonized Tanganyika (present Tanzania). Pig production for lard or bacon was an important consideration by the colonists and several breeding experiments were done with British pig breeds such as Large White, Yorkshire, Berkshire, Tamworth, and Large Black (Montgomery, 1921; Prosser, 1936). Pigs of the Large White breed imported from Kenya, as well as pigs distributed by the Ugandan veterinary department were kept by Ugandan farmers (Uganda, 1940). Details of the main breeds kept by the farmers are mostly lacking, but the pig populations in 1945 and 1959 were reported to be 23,158 and 15,668 (Masefield, 1962). Currently, pig production in Uganda is done by more than a million households that manage over 90% of the national herd of 4.2 million pigs (UBOS, 2019). Uganda’s per capita consumption is 3.4 kg/year (FAOSTAT, 2018) and the pro-poor significance of pig farming has recently attracted policy recognition (Sentumbwe, 2017).

While one study using microsatellite data has linked the genetic background of pigs in Uganda to European and Asian ancestries (Noce et al., 2015), the breed composition of most pigs in Uganda is largely unknown and any available breed information is mostly as reported by farmers. A previous study reported local pigs on smallholder farms in Uganda (Mbuza, 1995). According to Blench (2000), African pigs are usually black, with a straight tail and popped swept back ears. Other studies have mentioned exotic breeds like Hampshire, Large White, Duroc, Landrace, and Camborough^®^, which is a cross Large White, Landrace and Duroc, developed by the Pig Improvement Company (PIC^®^), having been introduced to Uganda (Ssewannyana and Mukasa, 2004; Walugembe et al., 2014; Greve, 2015; Roesel et al., 2016). Admixture between the different breed types is common.

Since 2012, he International Livestock Research Institute (ILRI) has provided a range of technical solutions to pig production constraints in districts of Uganda where pork production is important (Ouma et al., 2015). In 2017, the ILRI led Uganda Pig Genetics Project was launched to provide technical solutions to pig breeding constraints to support previous and ongoing initiatives. A key research question of the Uganda Pig Genetics project was to determine the most-appropriate pig breed or cross-breed type for different types of smallholder pig producers, considering a variety of issues including farmer preference and profitability, amongst others. As part of this work, household pig enterprises and the pigs within them were longitudinally monitored with genomic analysis undertaken to determine the breed-type of individual pigs kept. This study draws on this genomic data to quantify the genetic background, diversity and inbreeding levels of pigs on smallholder farms in Uganda using high throughput Single Nucleotide Polymorphism (SNP) data. SNP data from international sources, publicly available or privately owned but provided for this project, was used to place the pigs of Uganda onto a global genomic map.

## Materials and Methods

### Ethics Approval

This research was approved by the Institutional Research Ethics Committee (IREC), Institutional Animal Care and use Committee (IACUC) of the ILRI and Vector Control Division–Research and Ethics Review Committee (VCD-REC) of the Ministry of Health of Uganda (MOH). Prior informed consent was obtained from owners of the pigs sampled in Uganda. Research and access and benefit permits (Research Registration number: SS4550) were granted by Uganda National Council of Science and Technology (UNCST, 1990).

### Site and Household Selection

**FIGURE 1:**
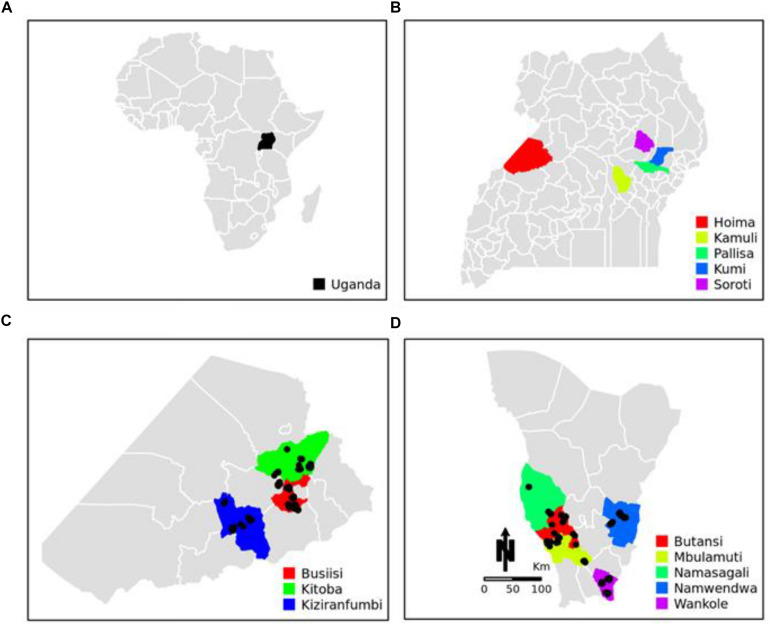
Geographical location of sampling sites. **(A)** Map of Africa showing the location of Uganda. **(B)** Map of Uganda showing the location of Hoima, Kamuli, Pallisa, Kumi, and Soroti districts. **(C)** Map of Hoima showing the locations of Busiisi, Kitoba, and Kizaranfumbi sub-counties. **(D)** Map of Kamuli showing the locations of Butansi, Mbulamuti, Namasagali, Namwendwa, and Wankole sub-counties. The black dots in panels **(C)** and **(D)** show the sampling locations.

This study was conducted in the districts of Hoima, Kamuli, Pallisa, Kumi, and Soroti in Uganda (Figures 1A–D). Hoima and Kamuli were the primary Uganda Pig Genetics project sites selected because of the importance of pig production to these districts, amongst other criteria. A purposively selected sample of 200 smallholder pig keeping households, 100 each from Hoima and Kamuli, participated in the study. The districts of Pallisa, Kumi, and Soroti were additional sampling sites for local Uganda pigs.

### Animals and Genotyping Data

**FIGURE 2:**
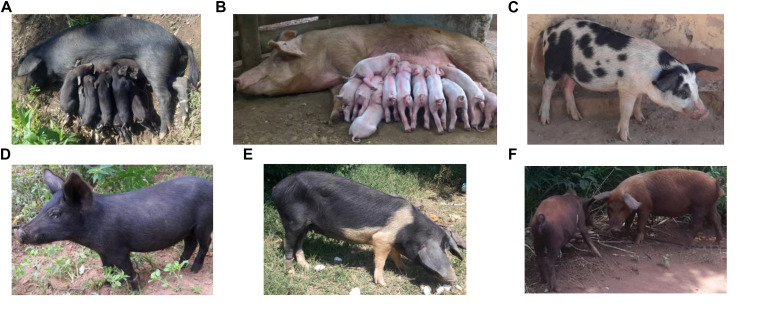
Photographs of pigs of different breed or cross-breed types in Uganda. **(A)** A local sow with her litter **(B)** an exotic breed e.g., Camborough^®^ sow with her litter **(C)** a spotted pig that could be old British (Gloucestershire) or a cross of exotic breeds (Landrace and/or Large White) **(D)** a local pig **(E)** a belted pig that could be a British Saddleback cross and **(F)** two red coated pigs that could be Duroc or Tamworth. Photo credit: Babigumira Brian Martin/ILRI/BOKU.

A sample of 422 pigs from the five districts in Uganda: Hoima (*n* = 163) Kamuli (*n* = 218), Kumi (*n* = 11), Pallisa (*n* = 12), and Soroti (*n* = 18) were involved in the study (see Figure 1). A total of 41 animals, showing the characteristics of African pigs according to Blench (2000), were sampled from 41 households having been reported to keep local Uganda pigs by extension staff in the latter three districts. Prevalence of pigs with black coat color, long snout, short legs and popped ears, facing up and backward were criteria of selection of households keeping local pigs. Hair samples taken from the 422 pigs were genotyped using the Neogen GeneSeek Genomic Profiler (GGP) Porcine 50K array (Neogen Europe, 2020). Using literature on East African pigs and phenotypic characteristics of pigs owned by the smallholder farmers in the study area (Figure 2), we chose as putative ancestral populations, Asian, Duroc, British, Iberian, and Continental European pig breeds. We explored the ancestry of Uganda pigs in global context by incorporating publicly or privately available genotypes from the putative ancestral populations (Cleveland et al., 2012; Yang et al., 2017; Pena et al., 2019; Pfeiffer et al., 2019; Hlongwane et al., 2020). The data were merged and manipulated in PLINK1.9 (Chang et al., 2015). Prior to merging the data, the SNP positions in each dataset were updated to the sus scrofa reference 11.1 genome build (Illumina, 2013). Quality control (QC) parameters were applied to exclude closely related individuals from each dataset based on PI_HAT using – genome and – max 0.1 flags. The PIC^®^ dataset consisted of 3359 animals. These were genotyped commercial animals – the Camborough^®^ a first filial generation (F1) cross between the PIC^®^ Landrace and PIC^®^ Large White pure lines genotyped with the Illumina PorcineSNP60 chip. The sample consisted of both male and female animals born since 2000 with varying degrees of kinship. Overall, the sampling technique avoided sampling multiple individuals from full-sib families (Cleveland et al., 2012). We use the code CMB throughout this paper to refer to the Camborough^®^ genotypes. The total genotyping rate for CMB data was around 15% lower than for other datasets, therefore we applied the –mind 0.15 flag to only this data. Data merging errors for SNPs with similar positions or on flipped strands were corrected using the – exclude or – flip flags. Samples were randomly excluded when a population exceeded 50. Also breeds without apparent interest to this study, according to literature, were excluded. The merged data (Table 1) was explored using Multidimensional scaling (MDS) and ADMIXTURE analysis (Alexander et al., 2009).

**TABLE 1:** Breeds/populations used in exploration of the ancestry of Uganda pigs.

Dataset	Breed/population	Country
Uganda Pig Genetics	Hoima, Kamuli, Pallisa, Soroti, and Kumi	Uganda
Cleveland et al., 2012	Camborough^®^ (Pig Improvement Company)	Great Britain
Pfeiffer et al., 2019; Hlongwane et al., 2020; Yang et al., 2017	Landrace and Large White	Austria, South Africa, and Denmark
Yang et al., 2017	Jinhua, Laihuwei, Lantang, and Meishan	China
	Angler Sattelschwein, Bunte Bentheimer	Germany
	Pietrain	Germany and Netherlands
	British Lop, Saddleback, Gloucestershire, Large Black, Leicester, Middle White, Tamworth, and Welsh	Great Britain
	Berkshire and Hampshire	Great Britain and United States
	Casertana and Nera Siciliana	Italy
	Poland Pulawska Spot	Poland
	Breitov, Livni, and Murom	Russia
Yang et al., 2017; Hlongwane et al., 2020	Duroc	America and South Africa
Pena et al., 2019	Entrepelado, Retinto, Entrepelado × Retinto cross, and Retinto × Entrepelado cross	Spain

### Multidimensional Scaling and ADMIXTURE Analysis

Following the exploratory admixture analysis outlined above, we narrowed down the list of reference populations to a panel that, to the best of our judgement, reflected the admixture seen in the Uganda population. This final reference panel included American (DRC), Chinese (MS), Spanish (IB), Modern European (CMB, LR and LW), Old British (SB and LB) breeds, and Local Ugandan pigs (LOC). We run MDS analysis on the merged dataset using the – distance-matrix flag of PLINK1.9 and Classical Metric MDS and plotted the MDS results in R (Team R Core, 2020). We also run unsupervised ADMIXTURE analysis on the merged dataset for number of ancestral population (K) from two to 10 (Alexander et al., 2009).

### Population Structure and Admixture Analysis Using CHROMOPAINTERv2, fineSTRUCTUREv4, and GLOBETROTTER

To support the ADMIXTURE and MDS analysis, we analyzed the data using the CHROMOPAINTERv2/fineSTRUCTUREv4 pipeline supported by the Perl scripts provided with the programs (Lawson et al., 2012). The data was phased using SAHPEIT2 (Delaneau et al., 2013). First, a custom R script (Team R Core, 2020) was run to prepare the genetic maps for each chromosome, as required by SHAPEIT2 based on the Sus scrofa recombination map (Tortereau et al., 2012). We run QC (–geno 0.2) and split the data by all autosomal chromosomes using PLINK1.9 (Chang et al., 2015). To achieve a successful run with the provided QC measures (considering size of individual populations and number of variants), we included the –force flag in the SHAPEIT2 command line. We run the impute2chromopainter.pl script to transform the SHAPEIT2 files into the phase format usable by Chromopianterv2. Next, we run the convertrecfile.pl script to generate recombination files using as inputs, the phase files from the previous run and genetic maps based on the Sus scrofa recombination map (Tortereau et al., 2012). We used the default settings for both scripts and specified the HapMap format when using the latter. We then run the phase and recombination files in CHROMOPAINTERv2 (Lawson et al., 2012) twice; the first run was to estimate nuisance parameters and the second one was to generate the co-ancestry matrix using the linked model. The Estimation-Maximization (E-M) iteration was run in automatic mode (“fs”) with the entire dataset for all autosomal chromosomes. Basically, each animal was conditioned on the others in 10 E-M iterations using a sample of ten animals. The main output were two inferred nuisance parameters (Ne, somewhat similar to effective population size and mu, the mutation/switch rate) (Hellenthal, 2012). These parameters (Ne = 34.7106 and mu = 0.00500584) were fixed in the CHROMOPAINTERv2 algorithm in the second run. The main outputs were estimation of the c-factor (effective number of chunks; *c* = 0.17931) and copying vectors. These outputs were fed into the Bayesian clustering algorithm of fineSTRUCTUREv4 for all autosomes.

To further investigate the admixture in the Ugandan pig population used in this study, we exploited the analytical capabilities of GLOBETROTTER (Hellenthal et al., 2014). The Bayesian clustering algorithm of fineSTRUCTUREv4 identified 40 clusters, which, when grouped, were generally not different from our labeled data or the output from ADMIXTURE1.3. Therefore, we run GLOBETROTTER to identify, date and describe admixture in the Uganda pigs using as surrogates: MS, DRC, IB, Modern European (CMB, LR, and LW) and Old British (SB and LB) and LOC with KAM or HOI as target (recipient) populations (Hellenthal et al., 2014; Hellenthal, 2020). We ran GLOBETROTTER with default settings for all parameters except “prop.ind,” “bootstrap.date.ind,” and “null.ind.” For the first run, we set “bootstrap.date.ind” to 0 and the other two to 1. In the second run, we set “prop.ind” to 0 and the other two to 1. For the third run, we set “null.ind” to 0 and the other two to 1 (Hellenthal, 2020). Here, we report the results from the last run.

### Autozygosity Analysis of Uganda Pigs

Autozygosity is the inheritance of alleles that are identical by descent (IBD). Contiguous homozygous genotype segments of the genome are called runs of homozygosity (ROH) (Gibson et al., 2006). The ROH can be used to infer the genomic inbreeding coefficient (F_*ROH*_) and distinguish ancient from recent inbreeding (Keller et al., 2011). We run this analysis using the dataset of Uganda (HOI and KAM) pigs (381 samples and 50,697 SNPs). The analysis was run in the cgaTOH (Zhang et al., 2013). The ROH analysis was run using minimum run lengths of 2, 4, 8, and 16 Mb and at least 20 SNP. Heterozygous calls in ROH were not allowed up to 16 Mb while only one heterozygous call was allowed for ROH > 16 Mb. The proportions of the ROH for each of the cut-offs (F_*ROHi*_, *i* = 2, 4, 8, and 16) were computed using as total length of autosome covered by SNPs of 2,259,445,079 bases. The genomic inbreeding coefficient (F_*ROH*_) was computed (McQuillan et al., 2008) as follows:

$$F_{\text{ROH}}=\sum \frac{L_{\text{ROH}}}{L_{\text{AUTO}}}$$

*L*_*ROH*_ is the sum of ROH per individual and *L*_*AUTO*_ is the total length of autosome covered by SNPs.

## Results

### Exploratory Analysis of Uganda Pigs in a Global Context

**FIGURE 3:**
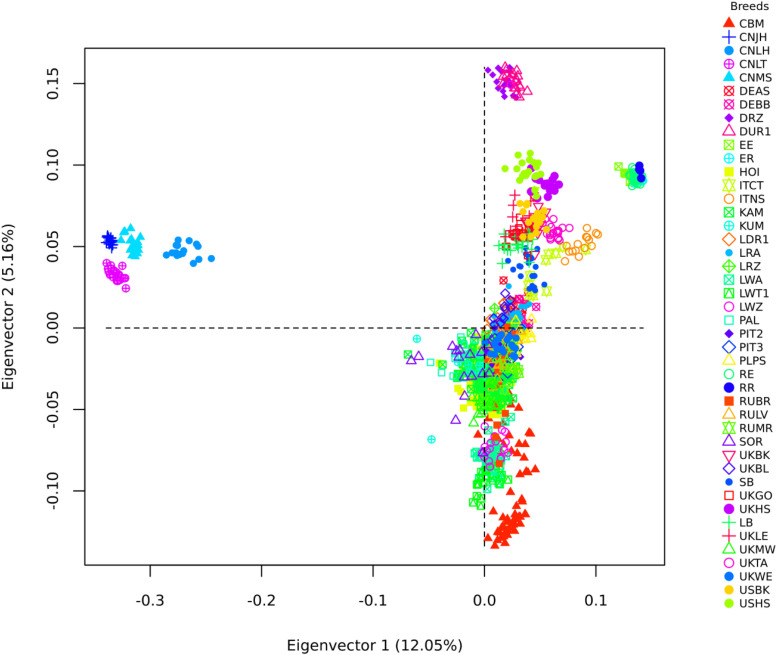
Multidimensional Scaling analysis of Uganda pigs in a global context. CMB: Camborough^®^; Great Britain; CNJH: Jinhua; CNLH: Laihuwei, CNLT Lantang; CNMS: Meishan-China; DEAS Angler Sattelschwein-Germany; DEBB Bunte Bentheimer-Germany; DRZ: Duroc-South Africa; DUR1: Duroc-United States; EE: Entrepelado; ER-Spain: Entrepelado × Retinto cross-Spain; HOI: Hoima-Uganda; ITCT: Casertana-Italy; ITNS: Nera Siciliana-Italy; KAM: Kamuli-Uganda; KUM: Kumi-Uganda; LDR1: Landrace-Denmark; LRA: Landrace-Austria; LRZ: Landrace-South Africa; LWA: Large White-Austria; LWT1: Large White-Denmark; LWZ: Large White-South Africa; PAL: Pallisa-Uganda; PIT2: Pietrain-German; PIT3: Pietrain-Netherlands; RE: Retinto x Entrepelado cross-Spain; RR: Retinto-Spain; RUBR: Breitov-Russia; RULV: Livni-Russia; RUMR: Murom-Russia; SOR: Soroti-Uganda; UKBK: Berkshire-Great Britain; SB: Saddleback-Great Britain; UKGO: Gloucestershire-Great Britain; UKHS: Hampshire-Great Britain; LB: Large Black-Great Britain; UKLE: Leicester-Great Britain; UKMW: Middle White-Great Britain; UKTA: Tamworth-Great Britain; UKWE: Welsh-Great Britain; USBK: Berkshire-United Sates of America; USHS: Hampshire-United Sates of America.

The merged dataset used in the exploratory analysis had 28,894 SNP**s** and 1,198 animals from 44 populations and 31 breeds (Table 2). The first eigenvector of the MDS analysis separated the Chinese and Iberian from the rest of the populations. The second eigenvector separated the Duroc from the rest of the populations. Both eigenvectors explained about 17% of the variation observed (Figure 3). The Ugandan samples were all situated inside a large cluster, including British and Continental European breeds.

**TABLE 2:** Ancestral and Uganda population used in ancestry analysis.

Dataset	Population	Breed	Code	Country	Samples
Cleveland et al., 2012	Modern European	Camborough^®^	CMB	Great Britain	30
Hlongwane et al., 2020	Duroc	Duroc	DRC	South Africa	20
Pena et al., 2019	Iberian	Iberian	IB	Spain	24
Uganda Pig Genetics	Hoima	Hoima	HOI	Uganda	161
	Kamuli	Kamuli	KAM		218
	Local	Local	LOC		38
Yang et al., 2017	Meishan	Meishan	MS	China	20
	Modern European	Landrace	LR	Denmark	20
		Large White	LW		16
	Old British	Saddleback	SB	Great Britain	20
		Large Black	LB		20

Following the exploratory analysis, we retained 30 of the 50 Camborough^®^ (CMB) samples based on proportions of both Large White and Landrace breeds. Further, we removed three local Ugandan pigs that had high exotic proportions. Finally, the panel of ancestral breeds narrowed down to those potentially interesting based on their ancestry contribution in the Uganda pigs. The final dataset (Table 2) had 28,894 SNPs, 587 samples from 9 populations and 7 breeds. The populations were Meishan, Duroc, Iberian, Modern European (Landrace, Camborough^®^, and Large White), old British (Saddleback and Large Black), Uganda (Hoima, Kamuli, and Local).

### Multidimensional Scaling and Admixture Analysis

**FIGURE 4:**
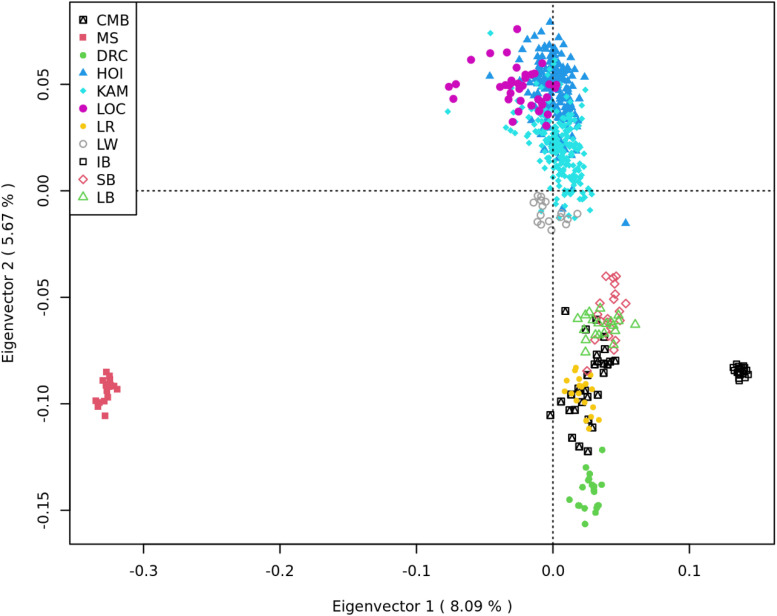
Multidimensional scaling analysis of Camborough^®^ (CMB); Meishan (MS); Duroc (DRC); Uganda [Hoima (HOI), Kamuli (KAM), and Local (LOC)]; Landrace (LR); Large White (LW); Iberian (IB); Saddleback (SB); and Large Black (LB). The first Eigen vector separates the MS and IB from the rest of the population. The second eigenvector closely clusters LW and Uganda pigs and separates them from the rest.

The first eigenvector of the MDS analysis of the dataset in Table 2 separates the Chinese and Iberian breeds from the Uganda, American, Modern European, and Old British breeds. The second eigenvector clusters some of the modern European breeds (largely comprised of Large White) closely with the Uganda pigs. It also separates the Uganda pigs from the rest of the Modern European, Old British, Duroc, Iberian and Chinese breeds (Figure 4).

**FIGURE 5:**
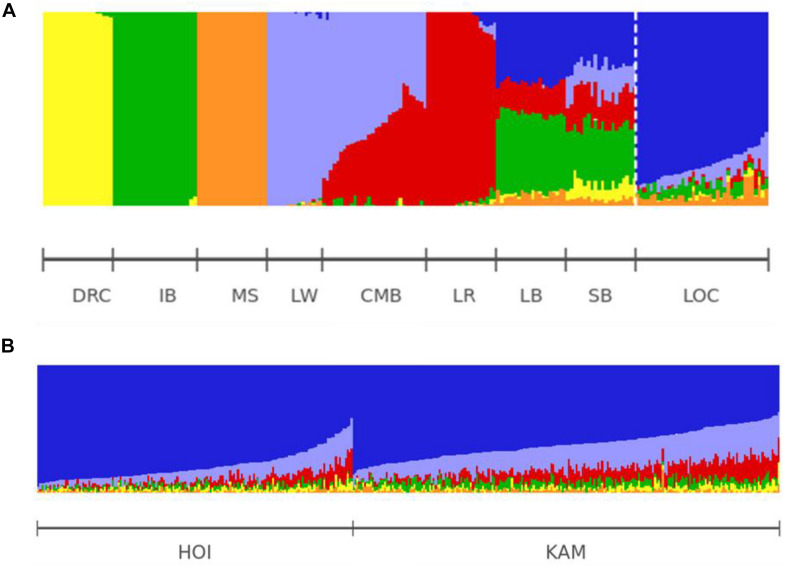
Admixture analysis. **(A)** Ancestry pig populations for *K* = 6: the modern European consists of Large white (LW; sky blue), Landrace (LR; red) and Camborough^®^ (CMB; nearly half sky blue/red). The old British breeds (SB: Saddleback: and LB: Large Black) share ancestries with the Iberian (IB), modern European and local Ugandan pigs (LOC). **(B)** The Uganda pigs Hoima (HOI) and Kamuli (KAM) have a dominant blue ancestry that we refer to as “local” shared with the old British breeds. The modern European breeds contribute most of the exotic ancestry.

We ran unsupervised analysis to infer ancestries of HOI and KAM pigs using various ancestral populations (K) without getting meaningful clusters at the lowest cross-validation error (CV) value. Therefore, we selected results at *K* = 6 and visualized the results using POPHELPER (Francis, 2017). The LOC pigs (purple) represented the main ancestry which was also shared with Old British breeds. The modern European breeds contributed most of the “exotic” ancestry in the Hoima and Kamuli pigs (Figures 5A,B).

Results from using ADMIXTURE1.3 showed that modern European breeds (CMB, LR and LW) contributed on average 22.8% with a range of 2–50% of the ancestry while LOC contributed 69.2% (36.9–95.2%). The other 8.0% were contributed by DRC, IB and MS. We also found higher frequency of MS ancestry in LOC than in HOI or KAM pigs (Figures 5A,B). Note that ADMIXTURE1.3 did not separate the Old British breeds into a uniform cluster but linked it to various populations, notably to Iberian and Ugandan types.

### Population Structure and Admixture Analysis Using CHROMOPAINTERv2, fineSTRUCTUREv4, and GLOBETROTTER

**FIGURE 6:**
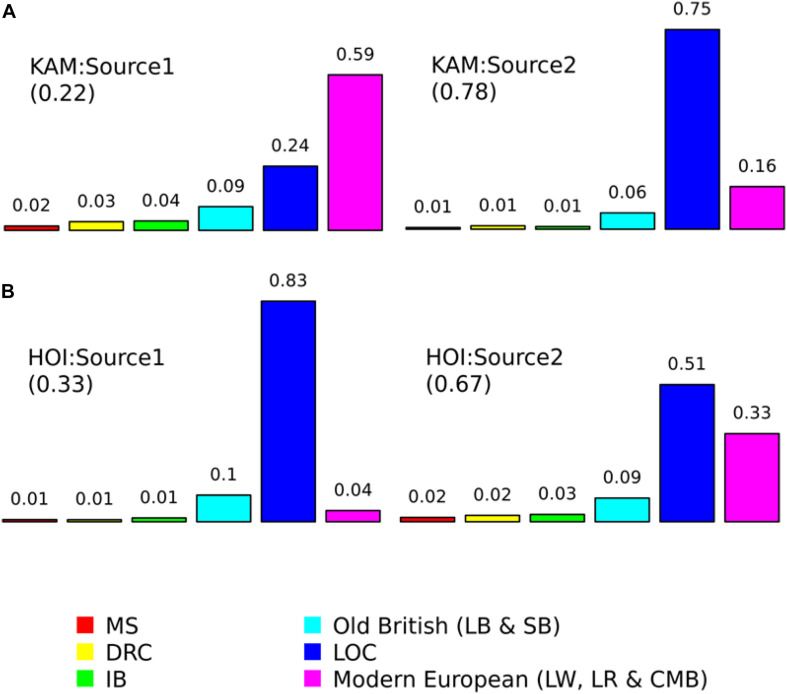
Proportions contributed by surrogate populations to the minor and major sources of admixture for **(A)** Kamuli (KAM) and **(B)** Hoima (HOI) pigs. The surrogate populations are: Meishan (MS); Duroc (DRC); Iberian (IB); Large Black (LB); Saddleback (SB); local Ugandan (LOC); Large White (LW); Landrace (LR); and Camborough^®^ (CMB). The numbers in brackets are the proportions each source contributes to the admixture in the target (recipient) population and they sum up to one. The numbers on top of the bars are the contributions of each surrogate population within each source and they sum up to one.

**FIGURE 7:**
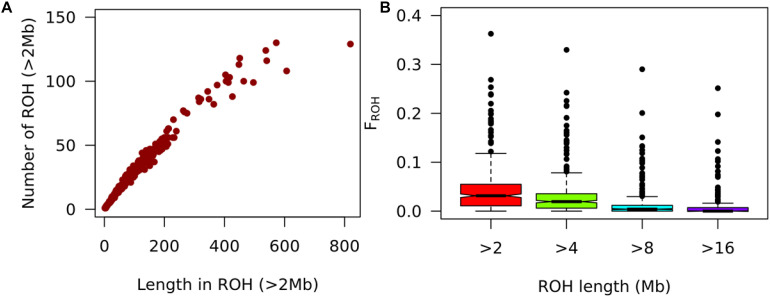
Analysis of autozygosity states. **(A)** Number of ROH distributed and along cumulative total ROH length on the pig genome in ROH. **(B)** Boxplot of_*FROH*_ cutoffs of 2, 4, 8, and 16 Mb for smallholder pigs from Hoima and Kamuli districts in Uganda.

The fineSTRUCTUREv4 analysis identifies three main clusters based on the empirical *c*-value – HOI, KAM, and LOC and the third cluster comprising international breeds (DRC, MS, IB, Modern European and Old British breeds). Considering that fineSTRUCTUREv4 did not identify clusters that differed much from our labeled data, we run “as is” the data in GLOBETROTTER to identify and date the admixture. GLOBETROTTER identified a one-date-multiway (1-DMW) for HOI and a one-date (1-D) admixture event with two sources for KAM pigs. The GLOBETROTER inferred date and confidence intervals (95% CI) for HOI and KAM were 6.371 (3.543-7.311) and 4.719 (2.420-5.093) generations (Figure 6). We converted generations to years using a generation interval of 1.9 years (Welsh et al., 2010) and the present year as 2019 in the formula (Hudjashov et al., 2017):

Y=y-(1+x)×g

where *Y* is the admixture date in years, *y*, the present year, *x*, the generations inferred by GLOBETROTTER and *g*, the generation interval. The admixture date and 95% CI (years) for HOI was 2004 (2003–2010) and for KAM, 2008 (2007–2012). For KAM, the best match sources of admixture were mostly Modern European (CMB, LR, and LW) and LOC pigs. The best match sources for the admixture event in HOI pigs were LOC and modern European (Figure 7).

### Autozygosity Analysis of Uganda Pigs

For a 50K SNP Chip, ROHs of length less than 2Mb may contain undetected heterozygotes and hence prone to false positives (Ferencakovic et al., 2013). Therefore, we reported inbreeding levels (F_*ROH* > 2Mb_) for ROH lengths greater than 2Mb. Only 348 of 381 pigs from Hoima and Kamuli districts, Uganda had at least one ROH > 2Mb. The F_*ROH* > 2Mb_ ranged from 0.000 to 0.363 with a mean of 0.043. The average ROH > 2Mb length per animal was 3.6 ± 1.9Mb and most pigs (81.6%) had at least one ROH > 4Mb. The average F_*ROH*_ for ROH length of 4, 8, and 16 were 0.030, 0.013, and 0.007. The longest individual ROHs (>20 Mb) were on chromosomes 4 and 14. The most inbred individual (*F*_*ROH* > 2Mb_ = 0.363) had 129 ROHs, the longest individual ROH (28.9Mb), longest total length of ROHs (819.35 Mb) for F_*ROH* > 2Mb_ and was from Kamuli district (Figures 7A,B).

## Discussion

### Breed Composition of Uganda Pigs

While we use the term LOC (local) to refer to pigs commonly considered to have been in Uganda for some time, we also note that no pig domestication event in Sub-Saharan Africa has been reported or supported by archeological or genetic evidence (Blench, 2000; Amills et al., 2013). Therefore, technically, indigenous Ugandan (Ugandese) pigs do not exist. However, we use the term local (LOC) throughout this paper to differentiate the resident population from exotic ones. We found what appeared LOC to relate more to ancestry contributions from black or belted old British pigs (Figures 5A,B). We also found signatures of MS in both the Uganda pigs as previously reported (Noce et al., 2015) and old British breeds. The MS signature in Uganda pigs is likely from an introgression through the old British breeds (Ramirez et al., 2009). We also observed a higher LOC ancestry in HOI than KAM pigs. This may be because Hoima is located further from Kampala, the capital of Uganda and a source of exotic pigs, than Kamuli. The local pigs of Uganda are not characterized and are only identified phenotypically according to the definition of African pigs by Blench (2000). It was difficult to find the local pigs especially where restocking programs had been or were operational. Our results complement previous findings and advocate for characterization and conservation of local pigs in Uganda.

The GLOBETROTTER analysis identified a one-date-multiway (1-DMW) admixture event for HOI pigs. The event involved mostly LOC and modern European breeds and dated the event to 2004 (95% CI: 2003–2010). In the case of Kamuli, a one date admixture event mostly involving modern European and LOC pigs was identified and dated to 2008 (95% CI: 2007–2012). These admixture dates imply recent introductions of exotic pigs in these areas, corresponding with varied “on-the ground” activities that have been observed with the introduction of new pig breeds. Pigs have been distributed to Ugandan farmers through programs run by the government and non-government organizations (Ampaire, 2011; Tatwangire, 2013; Ouma, 2017). The inferred admixture dates coincide with the out scaling of National Agricultural Advisory Services (NAADS) programs in Uganda. The NAADS program essentially sourced and distributed farm inputs including pigs and other livestock to smallholder farmers (Benin et al., 2007; Ouma et al., 2015). Non-government organizations in Kamuli that also distributed pigs to smallholder farmers are Volunteer Efforts for Development Concerns (VEDCO) (Ampaire, 2011), and Iowa State University-Center for Sustainable Rural Livelihoods (Csrl, 2021). Additionally, smallholder pig farmers in Uganda may also purchase pigs mainly from other nearby smallholder pig keepers or local markets (Ouma et al., 2015; Lichoti et al., 2016). These programs or farmers aim to improve productivity of pig herds through crossbreeding by distributing or purchasing pigs of commercial breeds including Landrace, Large White or Camborough^®^. The GLOBETROTTER and ADMIXTURE results together suggest the following. First, restocking programs have the potential to change the genetic constitution of smallholder pig herds. Second, the several admixture sources observed in the HOI and KAM pigs suggest indiscriminate crossbreeding (Greve, 2015) rather than for example a two- or three-way crossbreeding program. However, they could also suggest an ongoing upgrading of local herds given the proportionately higher frequency of the Modern European breed alleles.

### Inbreeding Levels of Uganda Pigs

Using the porcine GGP 50K SNP Chip, we investigated the occurrence of ROHs and quantified autozygosity in pigs in Kamuli and Hoima districts of Uganda. In this study, we found the genomic inbreeding coefficient (*F*_*ROH* > 2__*Mb*_) to be 0.043 (0–0.363) for HOI and KAM pigs. The low *F*_*ROH* > 2*Mb*_ indicates low inbreeding in the pig population. This is contrary to what has been previously reported (Tatwangire, 2014). Because of the very small herd size, sows are typically mated with village boars. Boar keepers usually source boars from outside the local area and the piglets received as payment for boar service are sold. Additionally, farmers with sows may source village boar service from sources outside their village depending on boar availability (Ouma et al., 2014; Lichoti et al., 2016). These scenarios suggest a low likelihood of mating related individuals, thus keeping inbreeding levels low. Somewhat higher inbreeding levels could be expected for households which own boars, but this a small minority. For instance, the most inbred individual (*F*_*ROH* > 2Mb_ = 0.363) could be the offspring of full sib or parent-offspring mating. Also, events necessitating stock replacement, like African Swine Fever outbreaks (Lichoti et al., 2016; Ouma et al., 2018) would also lower inbreeding levels.

## Conclusion

Smallholder pig production in Uganda is constrained by several factors, mostly related to pig health, nutrition and genetics (Ouma et al., 2015). Coupling genetic improvement with other appropriate management interventions would enhance productivity of smallholder pig herds. The results of this study showed that the contribution of Modern European ancestry did not exceed 50% for any of the animals sampled in Uganda. This was contrary to expectation, based on breed composition reported by smallholder farmers. The terms “local,” “crossbred,” and “exotic” used in this context seemed to reflect farmer perception rather than actual breed history. The gradient of ancestries observed in the Hoima and Kamuli populations of this study is still big enough to investigate the effect of the proportion of Modern European ancestry on growth, health and reproduction of pigs in those areas. Longitudinal data for these traits for most of the animals in the current study is available and will be analyzed subsequently. Only then appropriate crossbreeding levels may be determined and farmers advised about choice and sources of breeding stock.

## Data Availability Statement

The porcine 50k SNP chip data, including 50697 SNPs of 422 animals from Uganda included in this study was uploaded to DRYAD. The dataset has been assigned a unique identifier doi: 10.5061/dryad.4qrfj6q95 and is accessible via this temporary link: https://datadryad.org/stash/share/qKhv_9otEd2ivmo6TsIPuQHG30ZFg3BuSJjlg5SDj_M.

## Ethics Statement

The animal study was reviewed and approved by the Institutional Research Ethics Committee (IREC), Institutional Animal Care and use Committee (IACUC) of the International Livestock Research Institute (ILRI) and Vector Control Division–Research and Ethics Review Committee (VCD-REC) of the Ministry of Health of Uganda (MOH). Written informed consent was obtained from the owners for the participation of their animals in this study.

## Author Contributions

JS and KM conceived and designed the whole study. EO, JS, and KM provided the technical, administrative, and logistic support. BB oversaw the field-work, analyzed the data, and drafted the manuscript. CL and CP provided the genotype data through their companies. GM supported assembly of the data. BB and JS analyzed the data. GM, KM, CL, and CP contributed to the interpretation of the data. All authors critically revised and approved the manuscript for submission.

## Conflict of Interest

CP was employed by company PIG Austria GmbH. CL was employed by company Pig Improvement Company. The remaining authors declare that the research was conducted in the absence of any commercial or financial relationships that could be construed as a potential conflict of interest.
